# Analysis of the Influence of Process Parameters on the Properties of Homogeneous and Heterogeneous Membranes for Gas Separation

**DOI:** 10.3390/membranes12101016

**Published:** 2022-10-19

**Authors:** Daniel Polak, Maciej Szwast

**Affiliations:** Faculty of Chemical and Process Engineering, Warsaw University of Technology, Warynskiego 1, 00-645 Warsaw, Poland

**Keywords:** mixed matrix membranes, temperature dependence, pressure dependence, permeability, diffusivity, solubility

## Abstract

Heterogeneous membranes, otherwise known as Mixed Matrix Membranes (MMMs), which are used in gas separation processes, are the subject of growing interest. This is due to their potential to improve the process properties of membranes compared to those of homogeneous membranes, i.e., those made of polymer only. Using such membranes in a process involves subjecting them to varying temperatures and pressures. This paper investigates the effects of temperature and feed pressure on the process properties of homogeneous and heterogeneous membranes. Membranes made of Pebax^®^2533 copolymer and containing additional fillers such as SiO_2_, ZIF−8, and POSS-Ph were investigated. Tests were performed over a temperature range of 25–55 °C and a pressure range of 2–8 bar for N_2_, CH_4_, and CO_2_ gases. It was found that temperature positively influences the increase in permeability, while pressure influences permeability depending on the gas used, which is related to the effect of pressure on the solubility of the gas in the membrane.

## 1. Introduction

Membrane gas separation is a process increasingly used in industrial processes [1]. In many cases, it is displacing other, more classical gas separation processes, such as adsorption, absorption, or cryogenic treatment. Using membrane techniques, good results are obtained in the separation of air components [2,3,4], biogas components [5,6,7], the separation of helium from natural gas [8,9], hydrogen recovery [10,11,12], natural gas sweetening [13,14,15], or air dehydration [16] and natural gas dehydration [17,18]. In order to improve the efficiency of the membrane process and to optimize the energy consumption of such a process, single or multi-stage plants are used, along with recirculation of selected streams [19,20,21]. Process efficiency and performance are also affected by process conditions, in particular, operating pressure and temperature [22,23,24]. The very important aspect is the selection of the correct membrane for the specific application. It is the selectivity of the membrane towards selected components of the gas mixture and the permeability of the membrane that determines its suitability for a particular application. However, in this paper, we focus on the influence of operational conditions on membrane performance. We can imagine such processes where high pressure or high temperature are required. For example, the natural gas is obtained under a pressure of several dozen bar. Its purification with membranes could, therefore, take place at high pressures, without the need to reduce it first. In turn, synthesis gas is also obtained at a pressure of several dozen bar and, additionally, at a temperature of several hundred degrees Celsius. Its treatment by membranes could, again, take place with only partial cooling. Other such examples of elevated temperature gases are any flue gases. We should note that the membranes discussed in this paper are made of polymers, which naturally limits the pressures and temperatures at which they can be used.

In membrane gas separation processes, dense polymeric membranes perform best. However, it has been known for years that such membranes have their limitations, in that it is difficult to simultaneously achieve high membrane selectivity and high product flux [25,26,27]. A method for overcoming these limitations is the manufacturing of polymer-based heterogeneous membranes, also known in the literature as Mixed Matrix Membranes [28,29,30]. In such membranes, solid particles such as silica, organometallic structures, nanotubes, or nanowires are dispersed in a polymer matrix. The presence of these fillers in the polymer matrix causes changes in the physico-chemical properties of the material and, thus, affects the process properties (selectivity and permeability) of the membrane [31,32]. As a result of such changes, membranes made from polymers and fillers improve their process properties and are no longer subject to the same limitations as membranes made solely from polymer. This is the reason why there is a growing interest in the manufacture, research and use of heterogeneous membranes. The properties of heterogeneous membranes are influenced both by the type of filler used and by the concentration of this filler in the matrix. A number of works have been devoted to this issue. Membrane properties, as mentioned earlier, are also affected by process parameters such as temperature or feed pressure. This issue has received much less attention in the literature, which is the motivation for the research and analysis undertaken in this paper. Indeed, it can be found in the literature that operating conditions (pressure, temperature) affect the process [33,34,35,36,37,38]. However, these mentions in the literature are made as general remarks, or as remarks referring to the process as such or in the context of mathematical modelling. There is no mention in the literature regarding the influence of process conditions on the various parameters describing membrane properties. Understanding the relationship between the effect of process conditions on membrane properties will also allow for better planning of the membrane gas separation process.

This paper presents a study of the process properties of homo- and heterogeneous flat membranes made of block copolymer and three types of fillers, namely SiO_2_, ZIF−8, and POSS-Ph. The effects of temperature and feed pressure on membrane permeability and on diffusion and solubility coefficients were investigated.

## 2. Materials and Methods

### 2.1. Flat Membranes

The flat membranes were manufactured from a block copolymer with the trade name Pebax^®^2533 (Arkema, France). For heterogeneous membranes, the fillers were SiO_2_ nanoparticles (nanopowder 10–20 nm, Sigma-Aldrich, Poznan, Poland), ZIF−8 (Basolite Z1200 by BASF, Sigma-Aldrich, Poznan, Poland) or POSS-Ph (PSS-Octaphenyl-substituted, Sigma-Aldrich, Poznan, Poland). The rationale for undertaking research with these particular compounds and the structural formulae of these materials can be found in another paper of ours [31]. In that paper, material studies of such heterogeneous membranes are presented.

Preparation of the flat membranes started with dissolving the polymer granules in a solvent, which, in this case, was 2−butanol (Sigma-Aldrich, Poznan, Poland). In preparing homogeneous membranes, a 7 wt% solution of polymer in solvent was prepared each time. The weighed solvent and polymer were placed in an oil bath and stirred vigorously at 80 °C until the solution was completely dissolved and homogenized, which took at least 24 h. For the manufacture of heterogeneous membranes, weighed amounts of fillers, i.e., SiO_2_, ZIF−8, or POSS-Ph, respectively, were gradually added to the polymer solution at this stage. The amount of additives is specified as a mass percentage relative to the mass of the polymer in solution. When adding the fillers, the temperature of the solution was kept constant by constantly stirring it. The membrane-forming solution (mixture) was then left for a further 24 h at constant temperature and stirred continuously, this time at a lower intensity. In the case of homogeneous membranes, the step of adding fillers was omitted. Just before the membrane was formed, the solution was transferred to the ultrasonic bath for a few minutes. Finally, the solution was poured onto a heated glass plate and spread over it with a casting knife (Elcometer, Manchester, UK). The membrane thus prepared, while still in the liquid state, was left under controlled conditions until the solvent evaporated and the membrane solidified. The membrane was removed from the glass plate during the ultrapure water bath. After drying, the membrane was tested. 

The thickness of the fabricated membrane was measured by scanning electron microscopy (SEM) using a PhenomPro instrument (PhenomWorld, Eindhoven, The Netherlands).

### 2.2. Time Lag Method

The time lag method was used to measure membrane properties such as diffusion coefficient, solubility coefficient, or permeability [39,40]. A stand of our own design was used, which included a diaphragm module for a flat diaphragm, a vacuum pump, a connection to a gas pressure cylinder, pressure transmitters, a thermostatic device, and a computer with software.

The basic time lag method allows the diffusion coefficient (D) of the gas in the membrane to be determined. However, analysis of the rate of permeate pressure increase over time also allows the permeability of the membrane (P) to be determined, and consequently, by dividing the permeability by the diffusion coefficient, it allows the solubility coefficient (S) to be determined indirectly. This is consistent with Equation (1):

(1) P=S·D

The pure gases in vessels were supplied by Air Products (Warsaw, Poland).

## 3. Results and Discussion

The following chapter presents an analysis of the effects of feed pressure and process temperature on the separation properties of fabricated membranes with flat geometries. For this stage of the study, a homogeneous membrane made of Pebax^®^2533 was used. Two membranes with different concentrations of each filler used were selected, namely SiO_2_, ZIF−8, and POSS-Ph. Time-lag tests were performed for three different temperatures (25 °C, 45 °C, 55 °C) and three different feed pressures (2 bar, 4 bar, 8 bar). Pure gases of N_2_, CH_4_, and CO_2_ were used. 

**Figure 1:**
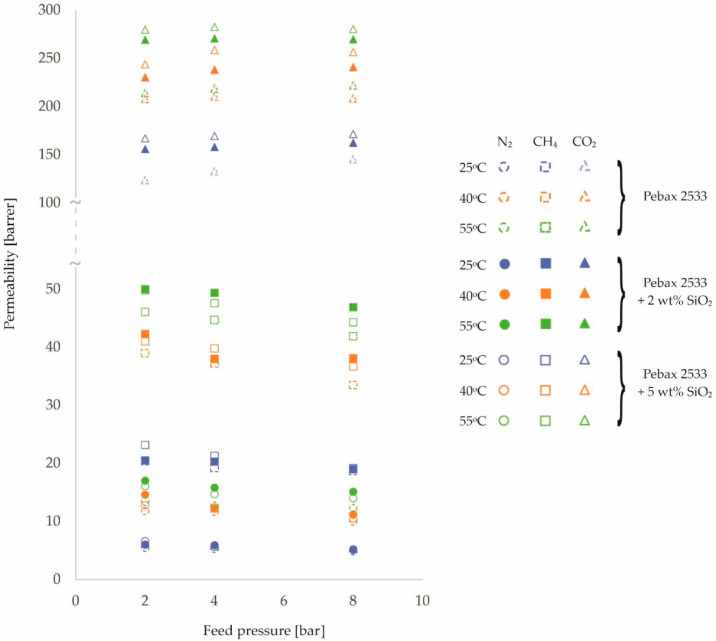
Change in permeability values of homogeneous membrane and heterogeneous membranes containing SiO_2_ for different feed pressure and temperature values.

**Figure 2:**
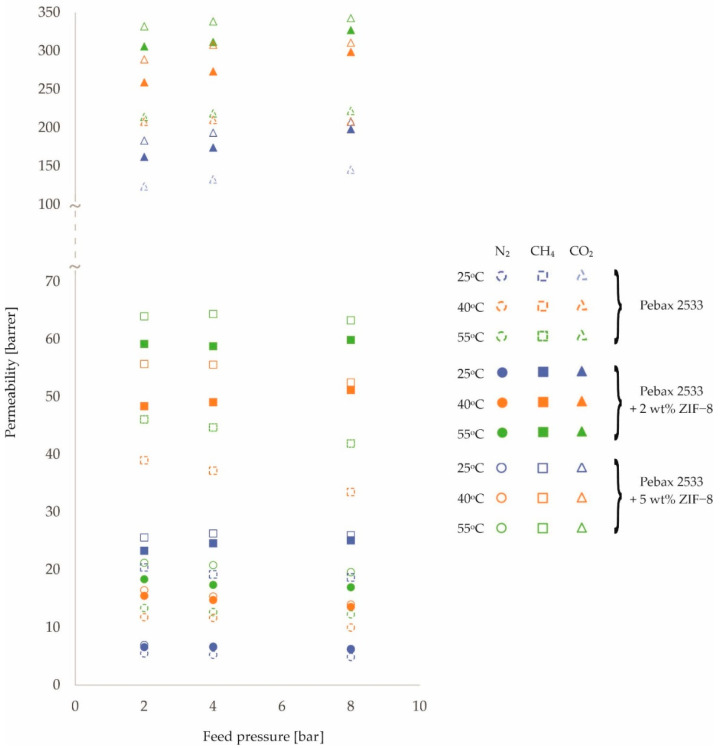
Change in permeability values of homogeneous membrane and heterogeneous membranes containing ZIF−8 for different feed pressure and temperature values.

**Figure 3:**
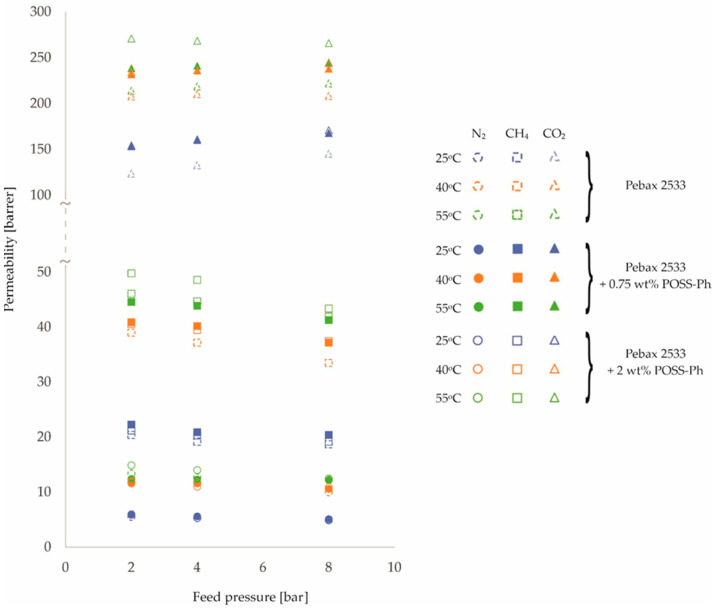
Change in permeability values of homogeneous membrane and heterogeneous membranes containing POSS-Ph for different feed pressure and temperature values.

The resulting permeability values are shown in Figure 1, Figure 2 and Figure 3 and in Table A1, Table A2, Table A3 and Table A4. In presenting the results of the gas permeation measurements of the developed membranes, a barrer unit was used, which is a non-SI unit, but is generally accepted in membrane-related literature. The conversion into SI units can be done as follows:

(2) $$1\;\mathrm{barrer}=3.35\;\cdot 10^{-16}\;\frac{\mathrm{mol}\;\cdot \;m}{m^{2}\;\cdot \;s\;\cdot \;\mathrm{Pa}}$$

An analysis of Figure 1, Figure 2 and Figure 3 and the corresponding Table A1, Table A2, Table A3 and Table A4 allows the following preliminary observations to be noted. The effect of feed temperature on membrane permeability is clearly discernible. Higher permeability values are observed for higher temperatures. This is consistent with the van’t Hoff–Arrhenius equation [41,42]. Furthermore, the mobility of the polymer chains increases with increasing temperature [43,44]. As a result, the transport of gas molecules across the membrane is facilitated. However, it should be noted that the magnitude of the variation in membrane permeability over the tested temperature range of 25 °C–55 °C depends on the gas and the filler used. The largest percentage increase in permeation with increasing temperature was obtained for N_2_, followed by CH_4_ and CO_2_. This relationship was obtained for all membrane types tested. In turn, for different fillers, the largest percentage increase in permeation as a function of temperature was obtained for ZIF−8 (ca. 40%), followed by SiO_2_ (ca. 27%) and POSS-Ph (ca. 22%) in comparison to the homogenous membrane.

In contrast, changes in permeability values as a function of pressure are not so clear-cut. For N_2_ and CH_4_ gases, permeability decreases slightly with increasing pressure, while for CO_2_ gas the situation is the opposite or, at the very least, no effect of pressure on the change in permeability is observed. Such observations can be made for both homogeneous and heterogeneous membranes. Explaining this effect requires further analysis, in particular, investigating the effect of pressure on diffusion and solubility coefficients. However, it is known that CO_2_ molecules, due to the fact that they possess a quadra-pole moment, can interact differently with polymer chains than molecules of other gases [45,46,47]. The effect observed for N_2_ and CH_4_ gases, i.e., a decrease in permeability with increasing pressure, can be explained by the compression of the polymer chains of which the membrane is made [24,48,49]. The trend lines for the individual pressure-dependent permeability variations, not drawn on the graphs in Figure 1, Figure 2 and Figure 3, are virtually parallel for a given gas and constant temperature. This means that the presence of nanoparticles filling the polymer matrix in a heterogeneous membrane has no additional effect on the compression (or prevention thereof) of the polymer chains in the membrane.

By analyzing Figure 1, Figure 2 and Figure 3, it is also possible to see the effect of the presence and concentration of fillers in the polymer matrix of the membrane on permeability. This issue is well known and described in the literature and will not be considered in this paper. 

**Figure 4:**
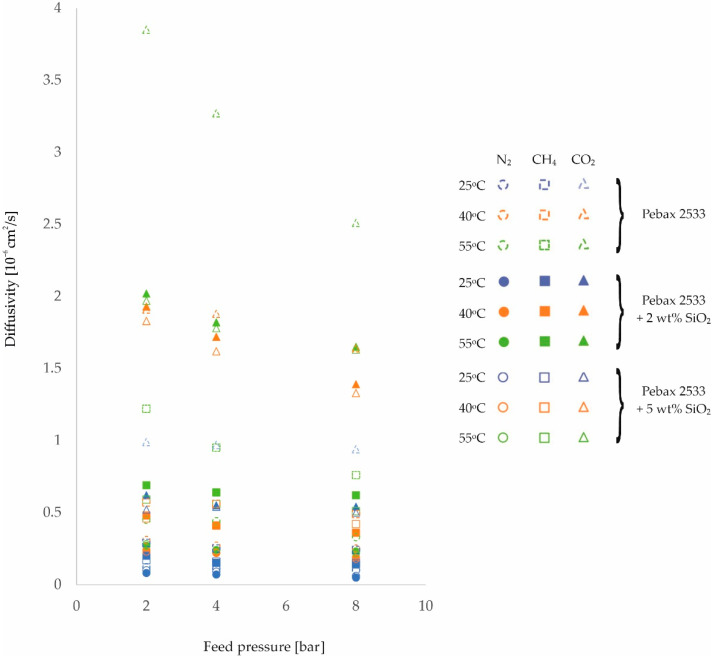
Change in diffusion coefficient values of homogeneous membrane and heterogeneous membranes containing SiO_2_ for different feed pressure and temperature values.

**Figure 5:**
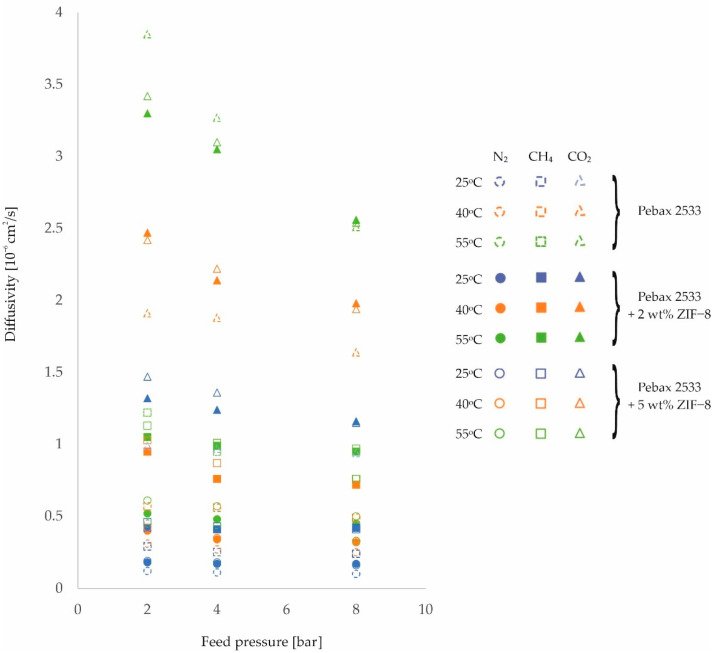
Change in diffusion coefficient values of homogeneous membrane and heterogeneous membranes containing ZIF−8 for different feed pressure and temperature values.

**Figure 6:**
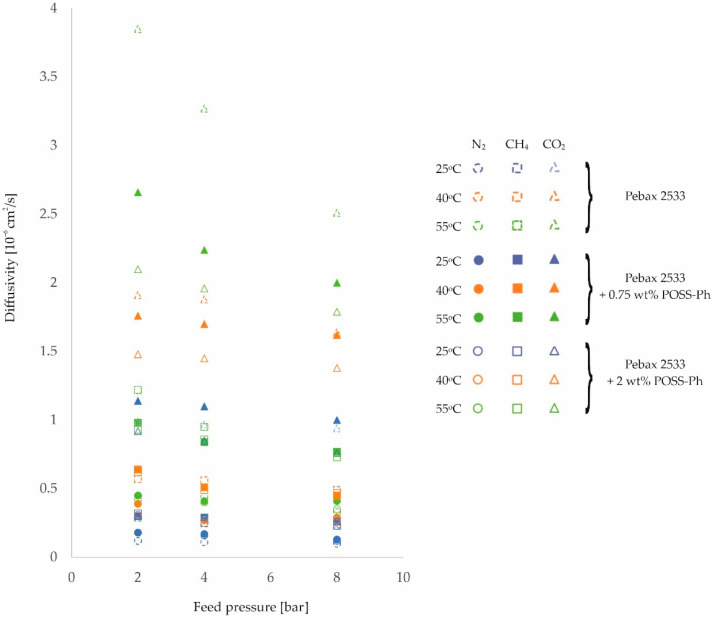
Change in diffusion coefficient values of homogeneous membrane and heterogeneous membranes containing POSS-Ph for different feed pressure and temperature values.

Further analysis of the effects of temperature and feed pressure on membrane properties will look at changes in the gas diffusion coefficient through homogeneous membrane and heterogeneous membranes. For the purposes of analysis, Figure 4, Figure 5 and Figure 6 and the corresponding Table A5, Table A6, Table A7 and Table A8 have been drawn up, which contain the values of the diffusion coefficients measured for different membranes, different gases, and at different process parameter values.

On the basis of these diffusion coefficient measurements, it can be concluded that for all of the gases tested, there is an improvement in their diffusivity with increasing temperature for all of the membranes tested. In addition, it can be observed that for heterogeneous membranes there is a lower percentage increase in diffusivity than is the case for homogeneous structures. This is related to the interactions between the filler particles and the polymer chains, which reduce the potential for their mobility to increase with increasing process temperature. This is particularly evident for CO_2_, but may be due to another adverse phenomenon. As the temperature increases, there is an increase in the free volumes in the polymer structure [50,51], which can result in an intensification of the contact between the filler particles and the polymer. The transport of this gas through the membrane is then impeded. It should also be noted that if the interactions described above were not present, the increase in gas diffusivity with temperature should be greatest for CO_2_. This is because the molecules of this gas have the smallest kinetic diameter of the gases studied [24,52].

In addition, from the values obtained for the diffusion coefficients, it can be seen that they decrease with increasing feed pressure for all membranes and gases tested. This effect is related to the compression of the polymer as a result of the applied pressure, as already mentioned. Thus, the distances between the polymer chains and their mobility are reduced, which adversely affects the transport stage of the molecules by diffusion. An analysis of the diffusion component of the ideal selectivity coefficient *α_Di/j_*, calculated as the quotient of the diffusivity coefficient of one gas and the diffusivity coefficient of the other gas, reveals no clear change in the value of this parameter and no unambiguous trend describing the influence of the feed pressure on this parameter. This means that the compression of the polymer due to the applied pressure limits the transport of the tested gases to a similar extent. Similarly, the presence of an inorganic additive does not affect the magnitude and trend of gas diffusion changes with feed pressure. 

**Figure 7:**
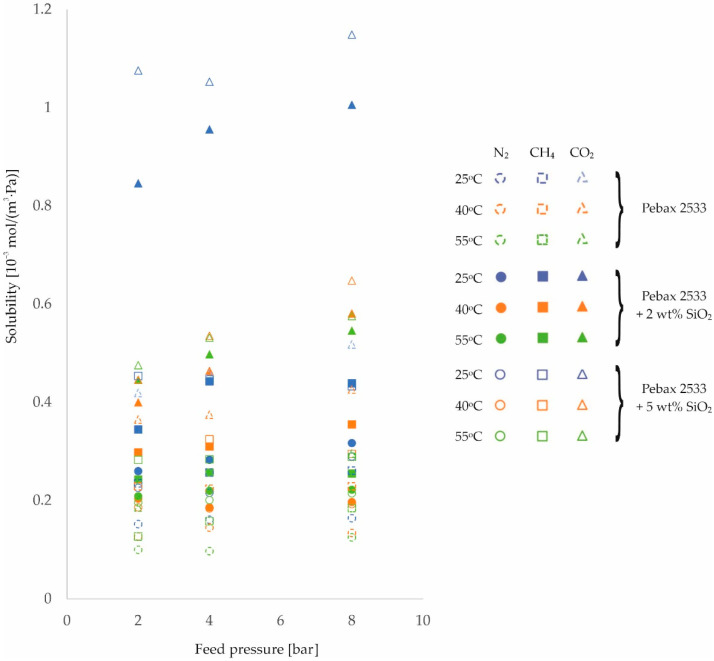
Change in solubility coefficient values of homogeneous membrane and heterogeneous membranes containing SiO_2_ for different feed pressure and temperature values.

**Figure 8:**
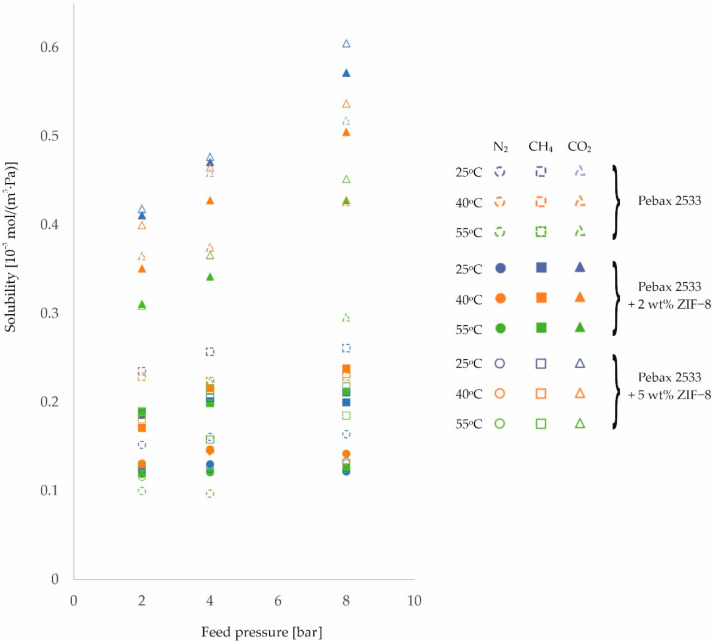
Change in solubility coefficient values of homogeneous membrane and heterogeneous membranes containing ZIF−8 for different feed pressure and temperature values.

**Figure 9:**
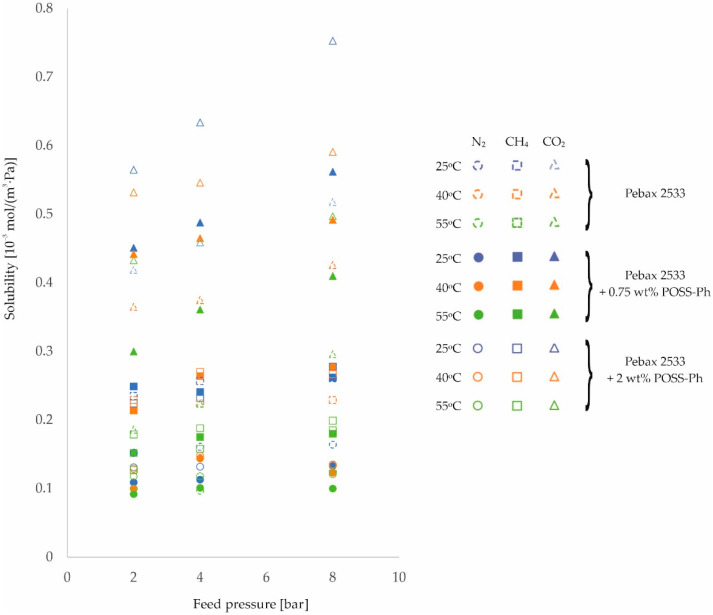
Change in solubility coefficient values of homogeneous membrane and heterogeneous membranes containing POSS-Ph for different feed pressure and temperature values.

The second parameter on which the rate of gas permeation through the membranes produced depends is the solubility coefficient, see Equation (1). The resulting values of this magnitude for different process conditions are shown in Figure 7, Figure 8 and Figure 9 and in Table A9, Table A10, Table A11 and Table A12, for the homogeneous membrane and heterogeneous membranes, respectively. It should be recalled at this point that the solubility values contained in this paper were determined as a quotient of the measured values of permeability and diffusion coefficient, and not determined in separate measurements.

From the results shown in Figure 7, Figure 8 and Figure 9, it can be seen that the solubility of gases on the surface of the fabricated materials decreases with increasing temperature. In contrast, as the feed pressure increases, the solubility of the gases in the membrane material increases. This is in line with predictions [53,54].

At this point, it is possible to return to the analysis of the effects of temperature and pressure on membrane permeability for CO_2_, which, for this gas, was characterized by a smaller effect of temperature and a different trend in the effect of pressure than for N_2_ and CH_4_ gases. 

Comparing the results obtained at 25 °C and 55 °C for the membranes tested, the following average percentage increases in permeability were recorded: for N_2_ +62%, for CH_4_ +57%, and for CO_2_ +40%. The markedly smaller increase in permeability for CO_2_ with increasing temperature is associated with a greater decrease in its solubility at the membrane surface (N_2_ −20%, CH_4_ −36%, CO_2_ −64%, respectively) and a smaller improvement in its diffusivity (N_2_ +67%, CH_4_ +67%, CO_2_ +62%, respectively) relative to the other gases with increasing temperature. The effects observed are related to the lower molar heat of condensation of CO_2_ relative to the other gases, and therefore also to the lower value of the enthalpy of dissolution, which additionally takes on a negative value for this gas [55]. This manifests itself in two effects. Firstly, a negative value of the enthalpy of dissolution means that the solubility of a given gas on the membrane surface deteriorates with increasing temperature, and secondly, the lower its value, the more difficult it is for the gas to condense [56]. This effect explains the different trend in changes in permeability with pressure to CO_2_ gas noted earlier.

**Figure 10:**
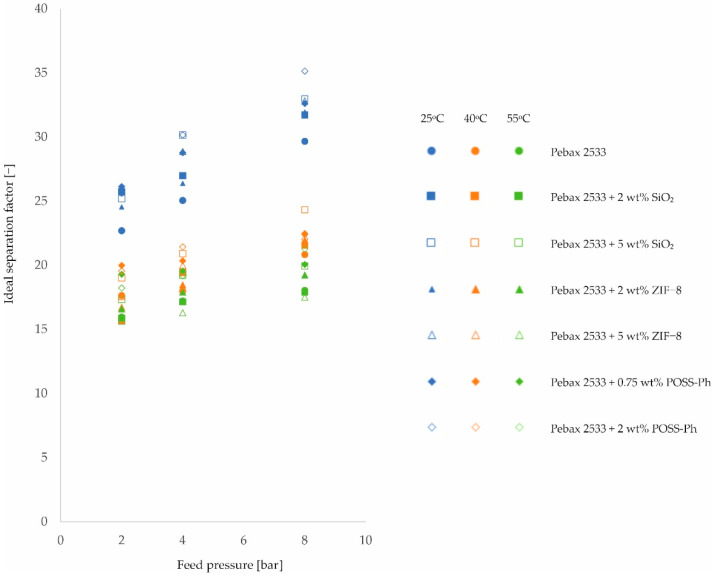
Change in ideal separation factor values for a N_2_/CO_2_ mixture of homogeneous membrane and heterogeneous membranes containing SiO_2_, ZIF−8, and POSS-Ph for different feed pressure and temperature values.

By dividing the permeability values for individual gases, the value of the ideal separation factor is obtained. Using the N_2_/CO_2_ mixture as an example, an analysis of the influence of process conditions on the ideal separation factor will be presented. The data shown in Figure 10 were obtained from the data shown in Figure 1, Figure 2 and Figure 3 and in Table A1, Table A2, Table A3 and Table A4.

Analyzing Figure 10, a strong correlation can be seen between the ideal separation factor and the feed pressure value. As the pressure increases, the ideal separation factor increases. This applies to all tested membranes, both homogeneous and heterogeneous. The trends for all these membranes are similar and the increase in the value of the ideal separation factor is approx. 40% when changing the feed pressure from 2 bar to 8 bar. A strong correlation is also observed when analyzing the influence of temperature on the value of the ideal separation factor. Here, however, the increasing temperature causes the value of the ideal separation factor to drop. The trends for the different types of membranes are similar. The decrease in the value of the ideal separation factor is ca. 30–45% (depending on type of filler) when changing the temperature from 25 °C to 55 °C. Physicochemical explanations of the observed effects should be sought in consideration of the influence of individual factors on the values of permeability, diffusivity, and solubility.

**Figure 11:**
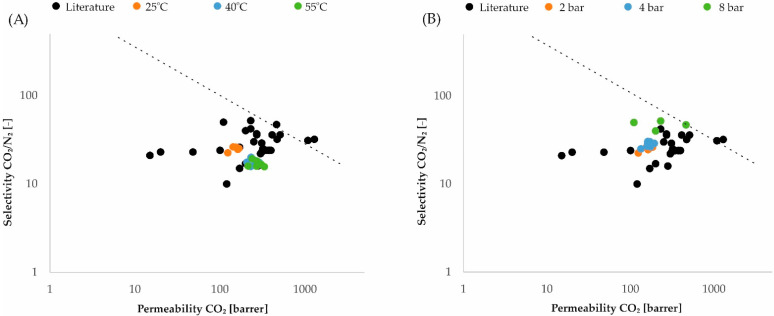
Comparison of results in the literature with the authors’ results: (**A**) temperature effect at constant pressure 1 bar; (**B**) pressure effect at constant temperature 25 °C.

In the literature, one can find many works devoted to research on gas permeability through membranes made of Pebax^®^2533 copolymer or its modifications [57,58,59,60,61,62,63,64,65,66,67,68,69]. Therefore, it may be interesting for the readers to compare the results in the literature and the results obtained in the research of this work on the Robeson charts [25,27]. Figure 11 shows the comparison of results in the literature obtained for the pure Pebax^®^2533 polymer and its modifications with the authors’ results, with the influence of pressure (Figure 11A) and temperature (Figure 11B) on the process parameters of the membranes. The considerations are limited to one gas mixture, namely the CO_2_/N_2_ mixture.

## 4. Conclusions

On the basis of the considerations presented above regarding the influence of process parameters on the diffusivity and solubility of gases, it is possible to determine the reasons for the changes in permeability of the membranes tested to the selected gases. 

The value of membrane permeability to gases is influenced by both diffusion coefficient and solubility. An increase in temperature positively increases diffusivity, but negatively affects solubility. An increase in pressure negatively increases diffusivity, but positively increases solubility. 

In general, an increase in temperature improves the permeability of membranes, meaning that the effect of temperature on diffusivity in this case outweighs the effect of temperature on solubility. In contrast, an increase in pressure worsens membrane permeability to N_2_ and CH_4_ gases, while it slightly improves permeability to CO_2_. For N_2_ and CH_4_ gases, the effect of temperature on diffusivity appeared to be greater than the effect of temperature on solubility. For CO_2_, on the other hand, the effect of temperature on solubility appeared to be the prevailing effect, which is due to the properties of the gas rather than the membrane.

## Data Availability

Not applicable.

## Appendix A

**Table A1:** Permeability of homogeneous membranes to different gases measured for different feed pressure and temperature values.

Membrane Type	Feed Pressure [bar]	Permeability *P_i_* [barrer]								
		N_2_			CH_4_			CO_2_		
		Process Temperature [ °C]			Process Temperature [ °C]			Process Temperature [ °C]		
		25	4[°C]	55	25	4[°C]	55	25	4[°C]	55
**PEBAX 2533**	2	5.5	11.8	13.4	20.4	39.0	46.1	123.7	207.9	214.0
		±0.1	±0.3	±0.3	±0.3	±0.6	±0.7	±1.0	±1.7	±1.7
	4	5.3	11.7	12.7	19.2	37.2	44.7	132.8	210.4	218.7
		±0.1	±0.3	±0.3	±0.3	±0.6	±0.7	±1.1	±1.7	±1.7
	8	4.9	10.0	12.3	18.7	33.5	41.9	145.4	208.4	222.0
		±0.1	±0.3	±0.3	±0.3	±0.5	±0.7	±1.2	±1.7	±1.8

**Table A2:** Permeability of heterogeneous membranes containing SiO_2_ to different gases measured for different feed pressure and temperature values.

Membrane Type	Feed Pressure [bar]	Permeability *P_i_* [barrer]								
		N_2_			CH_4_			CO_2_		
		Process Temperature [ °C]			Process Temperature [ °C]			Process Temperature [ °C]		
		25	4[°C]	55	25	4[°C]	55	25	4[°C]	55
**PEBAX 2533** **+2 wt% SiO_2_**	2	6.0	14.6	17.0	20.5	42.3	50.0	155.7	230.2	269.2
		±0.2	±0.4	±0.4	±0.3	±0.7	±0.8	±1.2	±1.8	±2.2
	4	5.9	12.2	15.8	20.3	38.0	49.4	158.0	238.1	270.6
		±0.1	±0.3	±0.4	±0.3	±0.6	±0.8	±1.3	±1.9	±2.2
	8	5.1	11.2	15.1	19.0	38.1	46.9	162.3	240.9	269.7
		±0.1	±0.3	±0.4	±0.3	±0.6	±0.8	±1.3	±1.9	±2.2
**PEBAX 2533** **+5 wt% SiO_2_**	2	6.6	12.8	16.1	23.2	41.0	49.8	167.0	243.8	279.8
		±0.2	±0.3	±0.4	±0.4	±0.7	±0.8	±1.3	±2.0	±2.2
	4	5.6	12.4	14.7	21.3	39.8	47.6	169.5	258.6	282.5
		±0.1	±0.3	±0.4	±0.3	±0.6	±0.8	±1.4	±2.1	±2.3
	8	5.2	10.5	14.0	19.2	36.7	44.3	171.5	256.4	280.4
		±0.1	±0.3	±0.4	±0.3	±0.6	±0.7	±1.4	±2.1	±2.2

**Table A3:** Permeability of heterogeneous membranes containing ZIF−8 to different gases measured for different feed pressure and temperature values.

Membrane Type	Feed Pressure [bar]	Permeability *P_i_* [barrer]								
		N_2_			CH_4_			CO_2_		
		Process Temperature [ °C]			Process Temperature [ °C]			Process Temperature [ °C]		
		25	4[°C]	55	25	4[°C]	55	25	4[°C]	55
**PEBAX 2533** **+2 wt% ZIF−8**	2	6.6	15.5	18.4	23.3	48.4	59.2	162.0	259.1	305.9
		±0.2	±0.4	±0.5	±0.4	±0.8	±0.9	±1.3	±2.1	±2.4
	4	6.6	14.8	17.4	24.6	49.1	58.8	174.2	273.3	311.6
		±0.2	±0.4	±0.4	±0.4	±0.8	±0.9	±1.4	±2.2	±2.5
	8	6.2	13.6	17.0	25.1	51.2	59.9	198.1	298.6	327.1
		±0.2	±0.3	±0.4	±0.4	±0.8	±1.0	±1.6	±2.4	±2.6
**PEBAX 2533** **+5 wt% ZIF−8**	2	7.0	16.5	21.2	25.6	55.7	64.0	183.3	289.0	332.0
		±0.2	±0.4	±0.5	±0.4	±0.9	±1.0	±1.5	±2.3	±2.7
	4	6.7	15.4	20.8	26.3	55.6	64.4	193.5	308.0	338.4
		±0.2	±0.4	±0.5	±0.4	±0.9	±1.0	±1.5	±2.5	±2.7
	8	6.3	14.0	19.6	26.0	52.5	63.3	207.7	310.8	342.7
		±0.2	±0.3	±0.5	±0.4	±0.8	±1.0	±1.7	±2.5	±2.7

**Table A4:** Permeability of heterogeneous membranes containing POSS-Ph to different gases measured for different feed pressure and temperature values.

Membrane Type	Feed Pressure [bar]	Permeability *P_i_* [barrer]								
		N_2_			CH_4_			CO_2_		
		Process Temperature [ °C]			Process Temperature [ °C]			Process Temperature [ °C]		
		25	4[°C]	55	25	4[°C]	55	25	4[°C]	55
**PEBAX 2533** **+0.75 wt% POSS-Ph**	2	5.9	11.6	12.4	22.3	40.9	44.6	153.5	232.0	238.5
		±0.1	±0.3	±0.3	±0.4	±0.7	±0.7	±1.2	±1.9	±1.9
	4	5.6	11.6	12.3	20.9	40.2	43.9	160.3	236.1	241.2
		±0.1	±0.3	±0.3	±0.3	±0.6	±0.7	±1.3	±1.9	±1.9
	8	5.1	10.6	12.2	20.4	37.2	41.3	167.8	238.0	244.9
		±0.1	±0.3	±0.3	±0.3	±0.6	±0.7	±1.3	±1.9	±2.0
**PEBAX 2533** **+2 wt% POSS-Ph**	2	6.0	12.0	14.9	21.2	40.6	49.8	154.5	234.6	271.0
		±0.2	±0.3	±0.4	±0.3	±0.7	±0.8	±1.2	±1.9	±2.2
	4	5.3	11.0	14.0	20.3	39.5	48.6	161.0	236.2	268.6
		±0.1	±0.3	±0.4	±0.3	±0.6	±0.8	±1.3	±1.9	±2.1
	8	4.9	10.9	12.5	19.2	37.5	43.4	171.1	244.0	266.0
		±0.1	±0.3	±0.3	±0.3	±0.6	±0.7	±1.4	±2.0	±2.1

**Table A5:** Diffusion coefficient of homogeneous membranes to different gases measured for different feed pressure and temperature values.

Membrane Type	Feed Pressure [bar]	Diffusivity *D_i_* [10^−6^ cm^2^/s]								
		N_2_			CH_4_			CO_2_		
		Process Temperature [ °C]			Process Temperature [ °C]			Process Temperature [ °C]		
		25	4[°C]	55	25	4[°C]	55	25	4[°C]	55
**PEBAX 2533**	2	0.12	0.31	0.45	0.29	0.57	1.22	0.99	1.91	3.85
		±0.01	±0.02	±0.03	±0.01	±0.04	±0.09	±0.06	±0.11	±0.22
	4	0.11	0.27	0.44	0.25	0.56	0.95	0.97	1.88	3.27
		±0.01	±0.02	±0.03	±0.01	±0.04	±0.07	±0.06	±0.11	±0.19
	8	0.10	0.25	0.33	0.24	0.49	0.76	0.94	1.64	2.51
		±0.01	±0.02	±0.02	±0.01	±0.03	±0.05	±0.05	±0.09	±0.14

**Table A6:** Diffusion coefficient of heterogeneous membranes containing SiO_2_ to different gases measured for different feed pressure and temperature values.

Membrane type	Feed pressure [bar]	Diffusivity *D_i_* [10^−6^ cm^2^/s]								
		N_2_			CH_4_			CO_2_		
		Process temperature [ °C]			Process temperature [ °C]			Process temperature [ °C]		
		25	4[°C]	55	25	4[°C]	55	25	4[°C]	55
**PEBAX 2533** **+2 wt% SiO_2_**	2	0.08	0.24	0.27	0.20	0.48	0.69	0.62	1.93	2.02
		±0.01	±0.02	±0.02	±0.00	±0.03	±0.05	±0.04	±0.11	±0.12
	4	0.07	0.22	0.24	0.15	0.41	0.64	0.55	1.72	1.82
		±0.00	±0.02	±0.02	±0.00	±0.03	±0.04	±0.03	±0.10	±0.1
	8	0.05	0.19	0.23	0.14	0.36	0.62	0.54	1.39	1.65
		±0.00	±0.01	±0.02	±0.00	±0.03	±0.04	±0.03	±0.08	±0.09
**PEBAX 2533** **+5 wt% SiO_2_**	2	0.10	0.23	0.28	0.17	0.46	0.59	0.52	1.83	1.97
		±0.01	±0.02	±0.02	±0.00	±0.03	±0.04	±0.03	±0.1	±0.11
	4	0.09	0.22	0.24	0.16	0.41	0.56	0.54	1.62	1.78
		±0.01	±0.02	±0.02	±0.00	±0.03	±0.04	±0.03	±0.09	±0.1
	8	0.06	0.18	0.22	0.15	0.42	0.51	0.50	1.33	1.63
		±0.00	±0.01	±0.02	±0.00	±0.03	±0.04	±0.03	±0.08	±0.09

**Table A7:** Diffusion coefficient of heterogeneous membranes containing ZIF−8 to different gases measured for different feed pressure and temperature values.

Membrane Type	Feed Pressure [bar]	Diffusivity *D_i_* [10^−6^ cm^2^/s]								
		N_2_			CH_4_			CO_2_		
		Process Temperature [ °C]			Process Temperature [ °C]			Process Temperature [ °C]		
		25	4[°C]	55	25	4[°C]	55	25	4[°C]	55
**PEBAX 2533** **+2 wt% ZIF−8**	2	0.18	0.40	0.52	0.42	0.95	1.05	1.32	2.47	3.30
		±0.01	±0.03	±0.04	±0.01	±0.07	±0.07	±0.08	±0.14	±0.19
	4	0.17	0.34	0.48	0.41	0.76	0.99	1.24	2.14	3.05
		±0.01	±0.02	±0.03	±0.01	±0.05	±0.07	±0.07	±0.12	±0.17
	8	0.17	0.32	0.45	0.42	0.72	0.95	1.16	1.98	2.56
		±0.01	±0.02	±0.03	±0.01	±0.05	±0.07	±0.07	±0.11	±0.15
**PEBAX 2533** **+5 wt% ZIF−8**	2	0.19	0.42	0.61	0.46	1.03	1.13	1.47	2.42	3.42
		±0.01	±0.03	±0.04	±0.01	±0.07	±0.08	±0.08	±0.14	±0.19
	4	0.18	0.35	0.57	0.43	0.87	1.01	1.36	2.22	3.10
		±0.01	±0.02	±0.04	±0.01	±0.06	±0.07	±0.08	±0.13	±0.18
	8	0.16	0.33	0.50	0.41	0.76	0.97	1.15	1.94	2.54
		±0.01	±0.02	±0.04	±0.01	±0.05	±0.07	±0.07	±0.11	±0.14

**Table A8:** Diffusion coefficient of heterogeneous membranes containing POSS-Ph to different gases measured for different feed pressure and temperature values.

Membrane Type	Feed Pressure [bar]	Diffusivity *D_i_* [10^−^^6^ cm^2^/s]								
		N_2_			CH_4_			CO_2_		
		Process Temperature [ °C]			Process Temperature [ °C]			Process Temperature [ °C]		
		25	4[°C]	55	25	4[°C]	55	25	4[°C]	55
**PEBAX 2533** **+0.75 wt% POSS-Ph**	2	0.18	0.39	0.45	0.30	0.64	0.98	1.14	1.76	2.66
		±0.01	±0.03	±0.03	±0.01	±0.04	±0.07	±0.06	±0.10	±0.15
	4	0.17	0.27	0.41	0.29	0.51	0.84	1.10	1.70	2.24
		±0.01	±0.02	±0.03	±0.01	±0.04	±0.06	±0.06	±0.10	±0.13
	8	0.13	0.29	0.41	0.26	0.45	0.77	1.00	1.62	2.00
		±0.01	±0.02	±0.03	±0.01	±0.03	±0.05	±0.06	±0.09	±0.11
**PEBAX 2533** **+2 wt% POSS-Ph**	2	0.18	0.31	0.42	0.32	0.62	0.93	0.92	1.48	2.10
		±0.01	±0.02	±0.03	±0.01	±0.04	±0.07	±0.05	±0.08	±0.12
	4	0.16	0.25	0.40	0.29	0.49	0.86	0.85	1.45	1.96
		±0.01	±0.02	±0.03	±0.01	±0.03	±0.06	±0.05	±0.08	±0.11
	8	0.12	0.27	0.35	0.23	0.47	0.73	0.76	1.38	1.79
		±0.01	±0.02	±0.02	±0.01	±0.03	±0.05	±0.04	±0.08	±0.10

**Table A9:** Solubility coefficient of homogeneous membranes to different gases measured for different feed pressure and temperature values.

Membrane Type	Feed Pressure [bar]	Solubility *S_i_* [10^−^^3^ mol/(m^3^·Pa)]								
		N_2_			CH_4_			CO_2_		
		Process Temperature [ °C]			Process Temperature [ °C]			Process Temperature [ °C]		
		25	4[°C]	55	25	4[°C]	55	25	4[°C]	55
**PEBAX 2533**	2	0.152	0.127	0.100	0.235	0.229	0.127	0.419	0.365	0.186
		±0.010	±0.008	±0.006	±0.007	±0.007	±0.004	±0.025	±0.022	±0.011
	4	0.161	0.145	0.097	0.257	0.224	0.158	0.459	0.375	0.224
		±0.010	±0.009	±0.006	±0.008	±0.007	±0.005	±0.028	±0.022	±0.013
	8	0.164	0.134	0.125	0.261	0.229	0.185	0.518	0.426	0.296
		±0.010	±0.008	±0.008	±0.008	±0.007	±0.006	±0.031	±0.026	±0.018

**Table A10:** Solubility coefficient of heterogeneous membranes containing SiO_2_ to different gases measured for different feed pressure and temperature values.

Membrane Type	Feed Pressure [bar]	Solubility *S_i_* [10^−^^3^ mol/(m^3^·Pa)]								
		N_2_			CH_4_			CO_2_		
		Process Temperature [ °C]			Process Temperature [ °C]			Process Temperature [ °C]		
		25	4[°C]	55	25	4[°C]	55	25	4[°C]	55
**PEBAX 2533** **+2 wt% SiO_2_**	2	0.260	0.204	0.209	0.345	0.298	0.243	0.846	0.400	0.446
		±0.016	±0.013	±0.013	±0.011	±0.009	±0.008	±0.051	±0.024	±0.027
	4	0.283	0.186	0.220	0.443	0.310	0.257	0.956	0.464	0.498
		±0.018	±0.012	±0.014	±0.014	±0.010	±0.008	±0.057	±0.028	±0.030
	8	0.317	0.197	0.222	0.439	0.355	0.255	1.006	0.581	0.546
		±0.020	±0.012	±0.014	±0.014	±0.011	±0.008	±0.06	±0.035	±0.033
**PEBAX 2533** **+5 wt% SiO_2_**	2	0.226	0.186	0.196	0.454	0.298	0.283	1.076	0.446	0.476
		±0.014	±0.012	±0.012	±0.014	±0.009	±0.009	±0.065	±0.027	±0.029
	4	0.216	0.184	0.201	0.447	0.325	0.284	1.053	0.536	0.532
		±0.014	±0.012	±0.013	±0.014	±0.010	±0.009	±0.063	±0.032	±0.032
	8	0.290	0.193	0.215	0.433	0.295	0.289	1.149	0.648	0.576
		±0.018	±0.012	±0.014	±0.013	±0.009	±0.009	±0.069	±0.039	±0.035

**Table A11:** Solubility coefficient of heterogeneous membranes containing ZIF−8 to different gases measured for different feed pressure and temperature values.

Membrane Type	Feed Pressure [bar]	Solubility *S_i_* [10^−^^3^ mol/(m^3^·Pa)]								
		N_2_			CH_4_			CO_2_		
		Process Temperature [ °C]			Process Temperature [ °C]			Process Temperature [ °C]		
		25	4[°C]	55	25	4[°C]	55	25	4[°C]	55
**PEBAX 2533** **+2 wt% ZIF−8**	2	0.123	0.130	0.119	0.186	0.171	0.189	0.411	0.351	0.311
		±0.008	±0.008	±0.007	±0.006	±0.005	±0.006	±0.025	±0.021	±0.019
	4	0.130	0.146	0.121	0.201	0.216	0.199	0.471	0.428	0.342
		±0.008	±0.009	±0.008	±0.006	±0.007	±0.006	±0.028	±0.026	±0.021
	8	0.122	0.142	0.127	0.200	0.238	0.211	0.572	0.505	0.428
		±0.008	±0.009	±0.008	±0.006	±0.007	±0.007	±0.034	±0.03	±0.026
**PEBAX 2533** **+5 wt% ZIF−8**	2	0.124	0.131	0.116	0.187	0.181	0.190	0.418	0.400	0.309
		±0.008	±0.008	±0.007	±0.006	±0.006	±0.006	±0.025	±0.024	±0.019
	4	0.125	0.147	0.122	0.205	0.214	0.214	0.477	0.465	0.366
		±0.008	±0.009	±0.008	±0.006	±0.007	±0.007	±0.029	±0.028	±0.022
	8	0.133	0.142	0.131	0.212	0.232	0.218	0.605	0.537	0.452
		±0.008	±0.009	±0.008	±0.007	±0.007	±0.007	±0.036	±0.032	±0.027

**Table A12:** Solubility coefficient of heterogeneous membranes containing POSS-Ph to different gases measured for different feed pressure and temperature values.

Membrane Type	Feed Pressure [bar]	Solubility *S_i_* [10^−^^3^ mol/(m^3^·Pa)]								
		N_2_			CH_4_			CO_2_		
		Process Temperature [ °C]			Process Temperature [ °C]			Process Temperature [ °C]		
		25	4[°C]	55	25	4[°C]	55	25	4[°C]	55
**PEBAX 2533** **+0.75 wt% POSS-Ph**	2	0.109	0.100	0.092	0.249	0.214	0.152	0.451	0.442	0.300
		±0.007	±0.006	±0.006	±0.013	±0.009	±0.009	±0.027	±0.026	±0.018
	4	0.113	0.144	0.101	0.241	0.264	0.175	0.488	0.465	0.361
		±0.007	±0.009	±0.006	±0.008	±0.007	±0.005	±0.029	±0.028	±0.022
	8	0.133	0.123	0.100	0.262	0.277	0.180	0.562	0.492	0.410
		±0.008	±0.008	±0.006	±0.008	±0.009	±0.006	±0.034	±0.03	±0.025
**PEBAX 2533** **+2 wt% POSS-Ph**	2	0.131	0.129	0.118	0.224	0.219	0.179	0.565	0.532	0.433
		±0.008	±0.008	±0.007	±0.007	±0.007	±0.006	±0.034	±0.032	±0.026
	4	0.132	0.148	0.118	0.232	0.270	0.188	0.634	0.546	0.459
		±0.008	±0.009	±0.007	±0.007	±0.008	±0.006	±0.038	±0.033	±0.028
	8	0.135	0.135	0.121	0.278	0.267	0.199	0.753	0.591	0.497
		±0.008	±0.008	±0.008	±0.009	±0.008	±0.006	±0.045	±0.035	±0.03
